# In vitro evaluation of antibacterial efficacy of vancomycin-loaded suture tapes and cerclage wires

**DOI:** 10.1007/s10856-021-06513-x

**Published:** 2021-04-06

**Authors:** Annette Eidmann, Andrea Ewald, Sebastian P. Boelch, Maximilian Rudert, Boris M. Holzapfel, Ioannis Stratos

**Affiliations:** 1 Department of Orthopaedic Surgery, Julius-Maximilians University Wuerzburg, Koenig-Ludwig-Haus, Brettreichstrasse 11, 97074 Wuerzburg, Germany; 2 Department for Functional Materials in Medicine and Dentistry, University Hospital Wuerzburg, Pleicherwall 2, 97070 Wuerzburg, Germany

## Abstract

Usage of implants containing antibiotic agents has been a common strategy to prevent implant related infections in orthopedic surgery. Unfortunately, most implants with microbial repellent properties are characterized by accessibility limitations during daily clinical practice. Aim of this in vitro study was to investigate whether suture tapes and cerclage wires, which were treated with vancomycin, show a sustainable antibacterial activity. For this purpose, we used 24 stainless steel wire cerclages and 24 ultra-high molecular weight polyethylene and polyester suture tape test bodies. The test bodies were incubated for 30 min. in 100 mg/ml vancomycin solution or equivalent volumes of 0.9% NaCl. After measuring the initial solution uptake of the test bodies, antibacterial efficacy via agar diffusion test with Staphylococcus aureus and vancomycin elution tests were performed 1, 2, 3, and 6 days after incubation. Vancomycin-loaded tapes as well as vancomycin-loaded cerclage wires demonstrated increased bacterial growth inhibition when compared to NaCl-treated controls. Vancomycin-loaded tapes showed an additional twofold and eightfold increase of bacterial growth inhibition compared to vancomycin-loaded wires at day 1 and 2, respectively. Elution tests at day 1 revealed high levels of vancomycin concentration in vancomycin loaded tapes and wires. Additionally, the concentration in vancomycin loaded tapes was 14-fold higher when compared to vancomycin loaded wires. Incubating suture tapes and cerclage wires in vancomycin solution showed a good short-term antibacterial activity compared to controls. Considering the ease of vancomycin application on suture tapes or wires, our method could represent an attractive therapeutic strategy in biofilm prevention in orthopedic surgery.

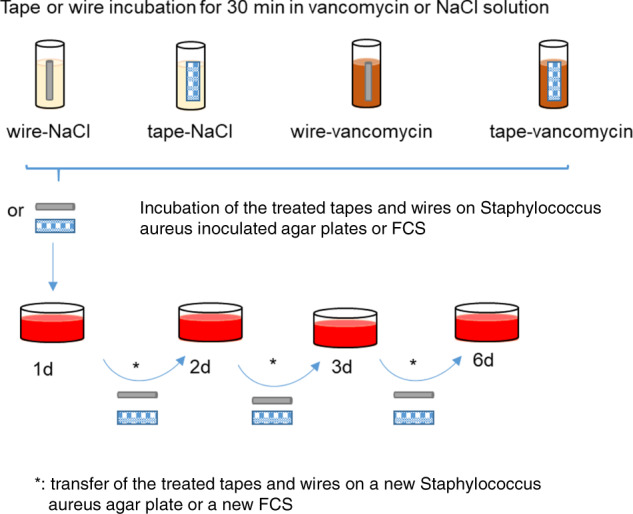

## Introduction

Implanted-related infections are considered as severe complications in orthopedic and trauma surgery with high social and economic impact. To prevent bacterial colonialization and biofilm formation and therefore an implant-related-infection, implants can be treated by active or passive surface coatings. Many surface modifications of commonly used implants have been tested and some gained translation into clinical practice [1–5].

In case of an implant-related infection, hardware removal and radical surgical debridement is the crucial step for achieving reduction of bacterial load [6]. Additionally, local application of antibiotic agents is widely used, to profit by increased local antibiotic concentration and decreased systemic side effects.

Although many implant-associated infections can be treated appropriately, there is often a conflict between the need of radical debridement and hardware removal on one hand and the need for stability and rigid fixation on the other hand. One common example that describes this conflict is the intraoperative femoral osteotomy for stem removal of an infected hip arthroplasty. To maintain at least some stability during the first weeks after osteotomy and removal of the implant, wire cerclages are often used to stabilize the osteotomy site despite newly implanted hardware can potentially lie ground for infection persistence or reactivation.

In that context, our purpose was to prevent the recurrence of infection by using cerclages with bacterial repellent properties. Instead of standard steel cerclage wires, we considered the use of suture tapes. Suture tapes are commercially available flat-braided non-absorbable sutures, which are usually used for tendon augmentation, especially for rotator cuff repairs [7, 8] but also for fracture or osteotomy osteosynthesis in special cases [9–11]. Consisting of ultra-high molecular weight polyethylene and polyester, they provide high strength and pressure distribution.

To achieve a bacterial repellent effect, we treated suture tapes and standard steel cerclage wires with a premixed vancomycin solution in a manner that can be performed easily during surgery in any operating theater. The aim of this study was to investigate whether vancomycin-loaded suture tapes and cerclage wires demonstrate any antibacterial activity in vitro.

## Material and methods

### Test bodies and test body preparation

As test bodies, we used 24 stainless steel cerclage wires, 1.3 mm in diameter (Synthes GmbH, Umkirch, Germany) or 24 ultra-high molecular weight polyethylene and polyester suture tapes (FiberTape^®^; Arthrex Inc., Naples, USA). Micrographs of both materials were taken using a stereo microscope (SteREO Discovery V20, Carl Zeiss, Göttingen, Germany). Each test body was 2.1 cm long. For the vancomycin group, 1 g vancomycin hydrochloride sterile powder (Hikma Farmaceutica, Terrugem, Portugal) was dispensed in 10 ml sterile physiologic NaCl (Fresenius Kabi, Bad Homburg, Germany) to obtain a vancomycin solution with a concentration of 100 mg/ml. For the control group, 0.9% NaCl (Fresenius Kabi, Bad Homburg, Germany) was used. Each test body was immersed in 1.5 ml of vancomycin solution or equivalent volume of NaCl for 30 minutes each at room temperature (24 °C). Before using the specimens for further testing, excess solution was removed by dabbing the test bodies on a sterile compress.

### Experimental groups

Four groups were formed: suture tapes incubated in vancomycin solution (tape-vancomycin), suture tapes incubated in sodium chloride (tape-NaCl) and cerclage wires incubated in vancomycin solution or sodium chloride (wire-vancomycin, wire-NaCl). Six test bodies were used per group for subsequent analysis at day 1, 2, 3, and 6 after incubation.

### Antibacterial efficacy via agar diffusion test

Agar diffusion test was used to test loaded suture tapes and cerclage wires for their antibacterial activity.

Staphylococcus aureus (S. aureus; clinical isolate) was grown over night in 10 ml LB-broth (2 g yeast extract, 4 g tryptone (both Applichem GmbH, Darmstadt, Germany), 2 g NaCl (Sigma-Aldrich, Germany) ad 400 ml H_2_O double dest) at 37 °C under aerobic conditions. 100 µl of this bacterial culture were spread onto LB-agar plates (LB-medium containing 1.5% (w/v) agar (Applichem GmbH, Darmstadt, Germany). The vancomycin- or NaCl-loaded test bodies were placed on one agar plate inoculated with S. aureus each and incubated at 37 °C for 24 h. After 24 h the test bodies were transferred onto a new agar plate inoculated with S. aureus to detect the remaining antibacterial activity. This procedure was repeated after 48 and 72 h. Pictures with a reference scale were taken of the samples before and after removal of the suture tape or the cerclage wire, respectively, using a digital camera. Photographic documentation was performed 1, 2, 3, and 6 days after incubation in vancomycin or NaCl solution.

The digital photographs were processed for planimetric analysis, using the open source image processing program ImageJ (ImageJ, NIH, Bethesda, Maryland, USA). The planimetric analysis was performed by two independent observers (AEi and IS). For further analysis the mean value of the collected data was used. After initial scaling, the visible zone of bacterial growth inhibition (quantified in mm²) and the surface of the test body (quantified in mm²) were measured. The relative zone of inhibition was calculated by dividing the zone of bacterial growth inhibition by the surface of the test body and multiplied by 100%.

### Elution tests

To analyze the kinetics of vancomycin release, 24 stainless-steel wire cerclages and 24 suture tapes were used and prepared as described in the section “Test bodies and test body preparation”. Each vancomycin- or NaCl-treated test body was placed in a test tube containing 3 ml fetal calf serum (FCS) (Bio & Sell, Feucht, Germany) and incubated at 4 °C for 24 h. After 24 h each test body was transferred into a new test tube containing 3 ml of clean FCS to analyze for further release capacity. This procedure was repeated after 48 and 72 h. Subsequent analysis was performed at day 1, 2, 3, and 6 after incubation with vancomycin or NaCl solution. A clinical analyzer (Indiko Plus, Thermo Fisher Scientific, Waltham, Massachusetts, USA) was used for enzyme immunoassay (range of measurement: 2.5–100 µg/ml). If vancomycin concentration exceeded the upper limit of measurement (100 µg/ml), the sample was diluted, measured again and the concentration was calculated.

### Absolute and relative solution uptake

To determine the amount of absorbed solution, test bodies were weighted before and after incubation with NaCl or vancomycin-solution. The weight measurements were performed by a precision balance (SBC 31, Scaltec Instruments, Göttingen, Germany). The difference in the weight before and after incubation was denoted as solution uptake and quantified in mg. The relative solution uptake was calculated as a fraction of the solution uptake divided by the length of each test body. The relative solution uptake indicates the amount of solution the test body is able to absorb per mm.

### Statistics

Results are shown as means ± standard error of the mean (S.E.M.). The statistical difference between the groups was calculated using one-way ANOVA for “solution uptake” and “relative solution uptake”. The statistical difference between the groups for the “zone of inhibition” and the “relative zone of inhibition” was calculated using two-way ANOVA followed by Tukey’s multiple comparison post hoc test. The level of statistical significance was defined as *p* < 0.05. A commercially available statistical software program (Prism 8, GraphPad, California, USA) was used for all statistical calculations.

## Results

### Zone of inhibition

**Fig. 1:**
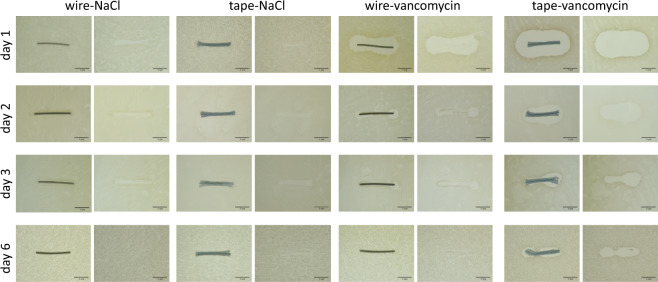
Agar diffusion test: the zones of inhibition are shown exemplarily for vancomycin loaded suture tape, vancomycin loaded cerclage wire, NaCl loaded suture tape and NaCl loaded cerclage wire on day 1, 2, 3, and 6 after incubation. Pictures demonstrate the agar plates before and after removal of the used test bodies (scale: 1 cm)

**Fig. 2:**
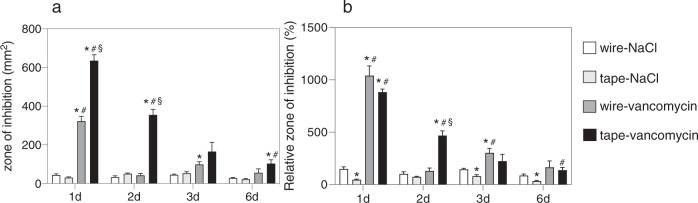
Planimetric analysis: efficacy of vancomycin or NaCl-loaded test bodies (suture tape or cerclage wire) measured by the zone of bacterial growth inhibition in mm² (**a**) and relative zone of bacterial growth inhibition in % to the surface of the coated test body (**b**). Suture tapes (tape) or cerclage wires (wire) were treated for 30 min with vancomycin solution or NaCl. Loaded materials were then incubated for 1 d, 2 d, 3 d, and 6 d on agar plates inoculated with Staphylococcus aureus. All data are given as means ± S.E.M.; *n* = 6 per time point and group; two-way ANOVA: *p < 0.05 versus wire-NaCl; ^#^*p* < 0.05 versus tape-NaCl; ^§^*p* < 0.05 versus wire-vancomycin

Vancomycin-treated tapes as well as vancomycin-treated cerclage wires showed bacterial growth inhibition at all time points, whereas within the control groups, a bacteria-free area could be detected only directly underneath the sample (Fig. 1). In both vancomycin groups (wire-vancomycin and tape-vancomycin) the zone of bacterial growth inhibition was significantly increased after 24 h when compared to NaCl-treated controls (*p* < 0.0001 for tape vancomycin vs NaCl-groups, *p* = 0.0002 for wire-vancomycin vs NaCl-groups) and then decreased continuously over time (Fig. 2a). The largest bacterial free surfaces could be observed for the tape-vancomycin group during all times points with a statistical significance compared to the wire-vancomycin group on day 1 and 2 (day 1: 635.9 ± 29.7 mm² vs 322.9 ± 24.4 mm², *p* < 0.0001; day 2: 355.3 ± 28.4 mm² vs 43.2 ± 9.2 mm², *p* = 0.0002) and on day 1, 2, and 6 compared to NaCl treated controls (*p* < 0.0001 day 1, *p* < 0.001 day 2, *p* < 0.05 day 3). Considering the relative zone of inhibition, a similar kinetic could be observed: vancomycin treated test bodies showed increased inhibition zones compared to NaCl treated test bodies at all time points with a significant difference at day 1 and 2 (Fig. 2b). Vancomycin treated tapes and wires presented comparably high values of bacterial growth inhibition at day 1, 3, and 6 and significant higher values for the tape-vancomycin group on day 2 (*p* = 0.0007 vs wire-vancomycin).

### Elution tests

**Fig. 3:**
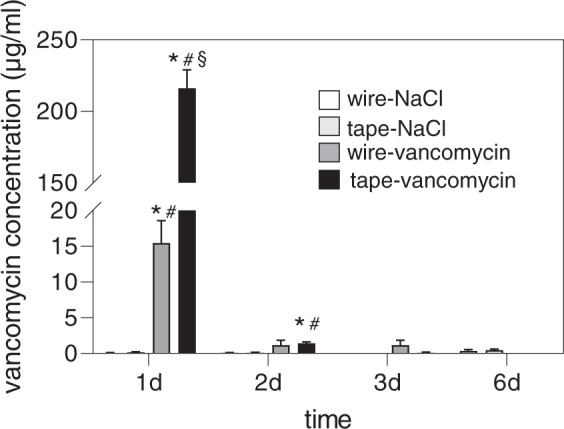
Elution test: vancomycin concentration (µg/ml) was measured in FCS after immersion with vancomycin or NaCl loaded test bodies (suture tape or cerclage wire) by enzyme immunoassay. Suture tapes (tapes) or cerclage wires (wire) were treated for 30 min with vancomycin solution or NaCl and then immersed in 3 ml FCS for 6 days. FCS was exchanged daily and taken for concentration measurements. All data are given as means ± S.E.M.; *n* = 6 per time point and group; two-way ANOVA: *p < 0.05 versus wire-NaCl; ^#^*p* < 0.05 versus tape-NaCl; ^§^*p* < 0.05 versus wire-vancomycin

Measurement of vancomycin concentration for the NaCl-treated tapes and wires showed low values ranging between 0 and 0.5 µg/ml, which were below the lowest limit of measurement of the analyzer. Vancomycin loaded wires reached a concentration up to 15.5 ± 3.1 µg/ml on the first day and decreased rapidly to low concentrations at day 2 and 3 after incubation (1.2 ± 0.7 µg/ml and 1.18 ± 0.66 µg/ml, respectively). At day 6, no vancomycin could be detected after eluting vancomycin treated wires. Vancomycin loaded suture tapes showed a high initial burst release on the first day (216.2 ± 12.8 µg/ml) and decreased rapidly on the second day (1.4 ± 0.2 µg/ml) (Fig. 3). The vancomycin concentration of the vancomycin-tape group was significantly higher on the first day compared to all other groups and time points (*p* < 0.0001).

### Capacity of solution uptake

The capacity of storing NaCl or vancomycin solution, respectively, was significantly higher for suture tapes than for cerclage wires (Table 1). The difference was significant for absolute solution uptake as well as for relative solution uptake.

**Table 1:** Solution uptake and relative solution uptake

	Solution uptake (mg)	Relative solution uptake (mg/mm)
Wire-NaCl	2.40 ± 1.38	0.10 ± 0.06
Tape-NaCl	9.53 ± 1.75*	0.40 ± 0.08*
Wire-vancomycin	7.10 ± 1.28*	0.28 ± 0.05*
Tape-vancomycin	15.78 ± 1.30*^#§^	0.68 ± 0.06*^#§^

Solution uptake (in mg) and relative solution uptake (in mg/mm) for the test bodies after immersion in vancomycin or NaCl for 30 min. All data are given as means ± S.E.M.; *n* = 6 per time point and group; one-way ANOVA: *p < 0.05 versus wire-NaCl; ^#^*p* < 0.05 versus tape-NaCl; ^§^*p* < 0.05 versus wire-vancomycin

## Discussion

In this in vitro study we demonstrated that suture tapes and steel cerclage wires, both loaded with vancomycin solution, showed antibacterial activity in agar diffusion tests. The antibacterial effect lasted up to 6 days and vancomycin-treated tapes showed larger overall-zones of inhibition than vancomycin-treated wires (Figs. 1 and 2). Regarding the vancomycin release kinetics of both materials, suture tapes reached a significant higher concentration level in FCS than wires within the first 24 h.

During the last decades, antibacterial coatings of surgical sutures have been the object of multiple research projects. As most sutures are used for wound closure, most of these studies focus on surface modifications of resorbable suture material, aiming to prevent surgical site infections [12–14]. In our study, we used non-absorbable braided suture tapes that provide high mechanical strength and can be applied instead of steel cerclages.

**Fig. 4:**
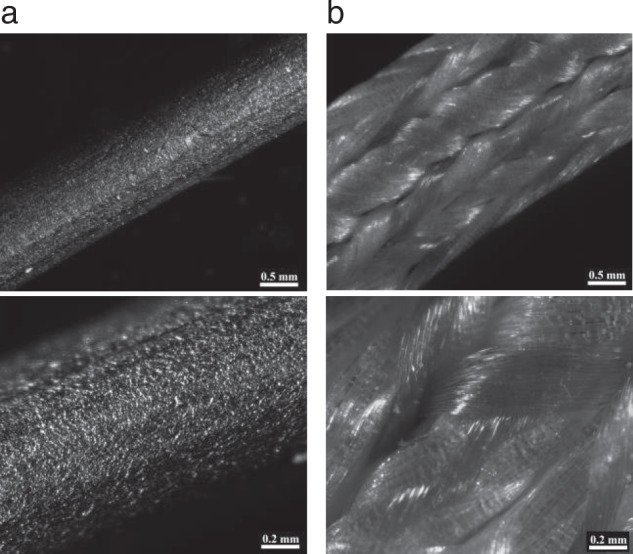
Micrographs of suture tape (**a**) and steel wire (**b**), showing the structural differences of both material: suture tapes consist of multiple, braided filaments, which lead to an enlarged surface, whereas steel wire shows only microscopic irregularities. Stereo microscopic, magnification: 40× (upper images) and 100x (lower images)

We showed that suture tapes were able to store significantly more agent, i.e., vancomycin sodium chloride or pure sodium chloride, per mm than steel wires. Although both materials are nonhygroscopic, capillary forces lead to adhesion of the aqueous test solution. Because of its braided structure, the surface of the suture tapes is enlarged; in addition, the antibiotic solution can remain between the suture filaments [15]. Figure 4 illustrates the different structure of both materials. The advantage of an increased vancomycin uptake results in a significantly higher drug concentration in serum elution tests. After changing the eluate after 24 h, serum concentrations fell rapidly to low concentrations. This might be explained by the good solubility of vancomycin sodium chloride in aqueous solution, which leads to an immediate release. In order to retard the drug release, the use of hardly soluble carriers like fatty acids have been investigated for suture coatings. Thereby, the drug release kinetics could be slowed down in dimensions that were depending on the carrier used, but as well showing the fastest release within the first 24 h [16–18].

Not only the preparation of the antibacterial solution, but also the coating process itself were more extensive in those studies and required more time and special material. In case of an intraoperative shaft fracture or osteotomy during septic implant removal and the subsequent need of fixation, our aim was to abstain from materials that per se could result in infection persistence and instead to use material that itself provide antibacterial effects. More importantly we were looking for techniques to equip these materials with antibacterial effect that would be easy to implement in a clinical setting. That means, that the material needed such as the antibiotic agents and the carrier solution, should routinely be stocked in the operation theater and that the preparation can be done in a relatively short time.

The local application of vancomycin powder into the operation situs, an even easier way of applying local antibiotics intending to prevent infection, has shown beneficial effects in spinal surgery concerning surgical site infections [19–21]. Looking at the prevention of implant related or periprosthetic infection, there is so far no evidence for the efficacy of local vancomycin powder application [22, 23].

DAC® (Novagenit Srl, Mezzolombardo, Italy), a hydrogel consisting of covalently linked hyaluronan and ploy-D,L-lactide, is approved for the European market for intraoperative implant coating [24]. By intraoperative mixing with different antibiotic agents, the hydrogel provides versatility and ease of handling and could be an alternative coating for suture tapes. Interestingly, elution tests with DAC^®^ showed similar release kinetics. After a peak release in the first 2–4 h, the release of the tested antibiotic compounds was almost complete or complete at 48 to 72 h [24]. In conclusion, a coating by simple immersion in vancomycin solution might be sufficient for fiber tapes.

In contrast to the fast decrease in vancomycin release in FCS, vancomycin-treated fiber tapes showed antibacterial efficacy during the whole testing period of 6 days using agar diffusion test. This result is comparable to other studies using this test setup for antibacterial coated sutures [15, 17, 24]. The discrepancy of antibacterial activity and antibiotic elution in our study might be explained by lacking larger amounts of daily exchanged aqueous solution on the agar plates. Boelch et al. demonstrated that the volume of immersion fluid influences the antibiotic elution of gentamicin/vancomycin loaded bone cements [25]. Daily complete exchange of the eluate simulates high wound fluid turnover, but in vivo these values can vary greatly dependent on the patient and the operation site and still remain an unpredictable variable [24, 25].

Measured vancomycin serum concentration on the first day for both vancomycin treated tapes and wires exceeded the minimal inhibitory concentration (MIC) of 2 µg/ml for S. aureus and of 4 µg/ml for most other susceptible pathogens [26]. Biofilm formation, a complex mechanism of adhesion of free-floating bacteria to the surface of the implant, followed by cell aggregation and biofilm maturation [3] is made responsible for most implant related infections. Immediately after bringing the implant into contact with the patients host tissue, host cells and pathogenic bacteria compete for adherence to the surface of the foreign body, what has been called the “race for the surface” [1, 27, 28]. The time window from the initial adhesion of bacteria to the irreversible stage of early biofilm formation provides the opportunity of therapeutic intervention. As the process of biofilm formation is considered to happen within the first 12–18 h [1, 29], the antibiotic burst release during the first 24 h shown in our study may be sufficient to avoid biofilm formation after implantation and therefore might be able to prevent early implant related infection. As a side effect, the short-term antibiotic release may reduce antibiotic resistance due to long lasting, insufficient antibiotic concentrations [24].

Our study has several limitations. In order to load the test bodies with the highest vancomycin concentration possible, we performed pre-tests to determine the solubility limit of vancomycin in sodium hydrochloride. For our study, we used this maximum concentration (100 mg/ml) for immersion. To verify the antibacterial efficacy, especially before the implementation into a clinical setting, different volumes and concentrations of vancomycin should be tested for coating. By using vancomycin as antibacterial drug, the spectrum of susceptible pathogens is limited to gram-positive bacteria, so that tests with other antibiotic substrates could be repeated.

From our study design it remains unclear, whether the antibiotic coating can resist mechanical stress. This is of particular interest when applying antibiotic loaded suture tapes in a clinical setting. Further testing is needed in this field. Whether the antibacterial effect and elution characteristics are the same in human tissue as in agar diffusion test and in FCS, could be examined in a clinical setting. When applying vancomycin loaded suture tapes in special, selected cases, tapes can be examined after explantation regarding biofilm formation and remaining antibacterial activity. Before using vancomycin-treated suture tapes in humans, the impact of the antibiotic substance on the mechanic properties of the suture tape has to be investigated, as well as its biocompatibility and cytotoxicity.

## Conclusion

After immersion in vancomycin sodium chloride, suture tapes as well as cerclage wires are bacterial repellent in a short-term setting. By reaching serum concentrations above the MIC in vitro, a beneficial effect on biofilm formation may exist, therefore providing an advantage in an infection situation. Further clinical evaluations are needed. The use of suture tapes treated by immersion in vancomycin solution remains an off-label use under responsibility of the operating surgeon.
